# Therapy program assisted with therapy dogs for children with Autism Spectrum Disorder (ASD)

**DOI:** 10.1192/j.eurpsy.2024.927

**Published:** 2024-08-27

**Authors:** A. Huguet Miguel, N. Cornudella Solans, P. Vergés Balasch, J. Bergadà Bell-lloc, V. Pera Guardiola

**Affiliations:** ^1^ Children and Adolescent Mental Health Service, Sant Joan de Deu Terres de Lleida; ^2^Ilerkan Association, Lleida, Spain

## Abstract

**Introduction:**

Autism spectrum disorders (ASD) is a neurodevelopmental disorder with an estimated lifetime prevalence of at least 1%. Some studies suggest that approximately 60% of children with ASD show emotional dysregulation and 44-86% sleeping disorders.

Studies suggest that cognitive behavioral therapy and animal-assisted therapy AAT can be an intervention strategy to promote emotional and behavioral dysregulation and sleep disturbances.

**Objectives:**

The purpose of this study was to investigate the effect of a therapy program assisted with dogs (AAT) together with cognitive behavioral therapy (CBT) on behavioral and emotional regulation and sleep disorders in children diagnosed with ASD.

**Methods:**

The sample was composed of 24 children between 7 and 10 years old diagnosed with ASD randomized into two groups (CGT group (control group) and AAT + CBT group (experimental group). Inclusion criteria: communication level of simple sentences, mild-moderate difficulties in behavioral and emotional regulation and sleep disturbances. Exclusion criteria: intellectual disability, children with specific dogs phobia. Assessment included ADOS-2, WISC-V, CGAS. dysregulation profile of Achenbach scale and Sleep Disturbance Scale for Children-Bruni. A program of 12 sessions (weekly one-hour sessions) focusing emotional and behavioral regulation and sleep disturbance was designed (Behavior Emotional Sleep Treatment Program. A pre-post evaluation was performed.

**Results:**

Participants were 20 boys and 2 girls (2 participants dropped out), with a mean age of 9. Regarding the socio-demographic and clinical characteristics, no significant differences has been observed between both groups in the global functioning measured with the Children’s Global Assessment scale (CGAS) (p=0.832), nor in the cognitive capacity (QI ) neither in reference to the associated comorbidities (p=0.103) nor in the variable prescription pharmacological treatment (p= 0.142). In emotional self-regulation, a significant improvement in emotional regulation difficulties was observed after treatment in both groups (experimental group: p=0.014; control group: p=0.012). However, the comparison between the pre-post intervention results between groups, regarding the emotional regulation variable, a greater improvement is observed in the experimental group (p=0.013). Significant improvements were also observed in sleep disorders (Bruni scale total score and in the sleep conciliation and maintenance difficulties scale) in both groups.

**Conclusions:**

To conclude, although this is a pilot study with a small sample size and further research is needed, results suggest that a therapy program assisted with therapy dogs and CBT have positive effects on emotional dysregulation and sleep disturbances in children with ASD and offers a possible intervention strategy.

**Disclosure of Interest:**

None Declared

